# Exploring the mucoadhesive behavior of sucrose acetate isobutyrate: a novel excipient for oral delivery of biopharmaceuticals

**DOI:** 10.1080/10717544.2019.1606866

**Published:** 2019-05-15

**Authors:** Stine Harloff-Helleberg, Lies A. L. Fliervoet, Mathias Fanø, Mechthild Schmitt, Maxim Antopolski, Arto Urtti, Hanne Mørck Nielsen

**Affiliations:** a Department of Pharmacy, Faculty of Health and Medical Sciences, University of Copenhagen, Universitetsparken 2, Copenhagen Ø, Denmark;; b Department of Pharmaceutics, Utrecht Institute of Pharmaceutical Sciences, Utrecht University, CG Utrecht, The Netherlands;; c Bioneer: FARMA, University of Copenhagen, Universitetsparken 2, Copenhagen Ø, Denmark;; d Division of Pharmaceutical Biosciences, Faculty of Pharmacy, Centre for Drug Research, University of Helsinki, Helsinki, Finland;; e School of Pharmacy, University of Eastern Finland, Kuopio, Finland

**Keywords:** Sucrose acetate isobutyrate, mucoadhesion, rheology, *in vivo* SPECT/CT, dynamic gastric model, biopharmaceuticals, oral drug delivery, insulin, mucoadhesion, Caco-2 cells

## Abstract

Oral drug delivery is an attractive noninvasive alternative to injectables. However, oral delivery of biopharmaceuticals is highly challenging due to low stability during transit in the gastrointestinal tract (GIT), resulting in low systemic bioavailability. Thus, novel formulation strategies are essential to overcome this challenge. An interesting approach is increasing retention in the GIT by utilizing mucoadhesive biomaterials as excipients. Here, we explored the potential of the GRAS excipient sucrose acetate isobutyrate (SAIB) to obtain mucoadhesion *in vivo.* Mucoadhesive properties of a 90% SAIB/10% EtOH (w/w) drug delivery system (DDS) were assessed using a biosimilar mucus model and evaluation of rheological behavior after immersion in biosimilar intestinal fluid. To ease readability of this manuscript, we will refer to this as SAIB DDS. The effect of SAIB DDS on cell viability and epithelial membrane integrity was tested *in vitro* prior to *in vivo* studies that were conducted using SPECT/CT imaging in rats. When combining SAIB DDS with biosimilar mucus, increased viscosity was observed due to secondary interactions between biosimilar mucus and sucrose ester predicting considerable mucoadhesion. Mucoadhesion was confirmed *in vivo*, as radiolabeled insulin entrapped in SAIB DDS, remained in the small intestine for up to 22 h after administration. Moreover, the integrity of the system was investigated using the dynamic gastric model under conditions simulating the chemical composition of stomach fluid and physical shear stress in the antrum under fasted conditions. In conclusion, SAIB is an interesting and safe biomaterial to promote high mucoadhesion in the GIT after oral administration.

## Introduction

Sucrose acetate isobutyrate (SAIB) is commonly used as an emulsion stabilizer in soft drinks and has been shown to remain in the gastrointestinal tract (GIT) for up to 24 h (Phillips et al., 1976), due to both limited absorption and degradation. SAIB is a sucrose derivative, obtained by full esterification of sucrose with acetate and isobutyrate groups, and it is generally regarded as safe (GRAS) by the U.S. Food and Drug Administration (FDA) (U.S. Food and Drug Administration, 2018). Interestingly, the potential of using SAIB has been studied in injectable drug delivery systems (DDSs) for small molecules (Lee et al., 2006; Lu et al., 2008; Wang et al., 2018). In those studies, SAIB was used to form a water insoluble, yet biodegradable, matrix when mixed with 10% ethanol resulting in a matrix from where the investigated drugs were released in a sustained manner. Also, a marketed SAIB-based product exists for long-acting injectable risperidone and bupivacaine formulations (Durect Corporation, 2016).

A noninvasive alternative to drug delivery by injection is oral administration (Renukuntla et al., 2013; Lam & Gambari, 2014). Successful oral delivery may, however, be greatly challenged by the low stability of drugs in the gastrointestinal (GI) tract, especially for peptides, proteins, and other biopharmaceuticals, which represents a growing part of the pharmaceutical portfolio (Hamman et al., 2005). Low stability is caused by the harsh luminal conditions including the presence of proteolytic enzymes and the significant variations in pH (Hwang & Byun, 2014). Moreover, the large molecular size of biopharmaceuticals limits their penetration into and through not only the mucus layer lining the epithelial surface but also the epithelial membrane. Much work has been done to overcome these obstacles, utilizing various strategies to increase the overall transmucosal absorption by employing absorption enhancers, enzyme inhibitors, and mucoadhesive polymers (Khafagy et al., 2007; Li et al., 2012; Hwang & Byun, 2014) in order to deliver intact pharmacologically functional molecules. Moreover, the focus has been on protecting the biopharmaceutical from being rapidly degraded when trafficking through the harsh GI environment by using advanced DDSs (Park et al., 2011; García-Díaz et al., 2015).

A growing interest among researchers exists regarding delivery via the GI mucosa (Boegh et al., 2013). One strategy is to increase the contact time between the DDS and target site for absorption (Ivarsson & Wahlgren, 2012; Reineke et al., 2013), hence improve the flux of the drug across the mucosal membrane and thus enhance its systemic bioavailability. Thus, SAIB is a highly relevant biomaterial to consider for oral administration of drugs due to (1) a proven prolonged residence time in the GIT (Reynolds & Chappel, 1998), (2) its capability to form a biodegradable matrix from where drugs can be released in a controlled manner (Lu et al., 2008; Jølck et al., 2014), and (3) its status as a GRAS excipient (Food Additive Status List, 2002). However, to the best of our knowledge, the mucoadhesive behavior of SAIB, hence potential as a non-injectable biomaterial, still remains to be studied.

Thus, the present work explored the potential of SAIB as a biomaterial to obtain mucoadhesion *in vivo*. First, rheological profiling of SAIB DDS was done at various temperatures, after incubation in biorelevant media and upon mixing with biosimilar mucus to assess the mucoadhesive behavior. Second, we studied the effect of physical shear stress on the SAIB DDS in the antrum using an advanced dynamic gastric model. Insulin was used as a model cargo to evaluate the capability of the SAIB DDS to retain a poorly stable drug when subjected to the harsh GI environment. As a control, release studies of insulin were done in both 10 mM MES buffer and simulated small intestinal fluid (SSIF) (both pH 6.5), and the release profile was related to both rheological properties of SAIB, hence microstructure during the experiment. Third, the mucoadhesive behavior of orally administered SAIB DDS loaded with ^123^I-labeled insulin was studied in F344/NCrHsd rats using SPECT/CT imaging. Prior to the *in vivo* studies, the effect on epithelial integrity and cell viability was evaluated *in vitro*.

## Materials and methods

### Chemicals and reagents

Human insulin was kindly provided by Sanofi-Aventis Deutschland (Frankfurt, Germany). 4-Morpholineethanesulfonic acid (MES) and 4-(2-hydroxyethyl)piperazine-1-ethanesulfonic acid (HEPES) were purchased from AppliChem (Darmstadt, Germany), polyacrylic acid (PAA) (Carbopol^®^ 974P NF) from Lubrizol (Brussels, Belgium), SAIB, trypan blue, Hanks’ balanced salt solution (HBSS), mucin from porcine stomach type II, bovine serum albumin (BSA) (98%), cholesterol (>99%), polysorbate 80, sodium taurocholate, sodium dodecyl sulfate, potassium dihydrophosphate, sodium thiosulfate, sodium chloride, potassium chloride, calcium chloride, monosodium phosphate, chloramine T, Dulbecco’s modified Eagle’s medium (DMEM), fetal bovine serum (FBS), penicillin/streptomycin, l-glutamine, and non-essential amino acids from Sigma-Aldrich (St. Louis, MO), soybean phosphatidylcholine and phosphatidylcholine (PC, purity 98%) from Lipoid (Ludwigshafen am Rhein, Germany), ^3^H-mannitol (1 mCi) and Ultima Gold scintillation fluid from PerkinElmer (Cleveland, OH), Na-^123^I from MAP MEDICAL (Tikkakoski, Finland), 3-(4,5-dimethylthiazol-2-yl)-5-(3-carboxymethoxyphenyl)-2-(4-sulfophenyl)-2H-tetrazolium (CellTiter 96^®^ AQ_ueous_ MTS Reagent Powder), and phenazine methosulfate (PMS) from Promega (Fitchburg, WI). All other chemicals used were of analytical grade and obtained from commercial sources. For RP-HPLC, solvents were HPLC-grade. Ultrapure water was used throughout the study and prepared using a Barnstead NanoPure system (Thermo Scientific, Waltham, MA).

### Preparation of test material and buffers

Based on previous studies (Okumu et al., 2002; Pechenov et al., 2004; Lu et al., 2007, 2008), SAIB DDS was prepared by adding 10% (w/w) ethanol (96%) to SAIB, allowing it to dissolve at 37 °C overnight. Subsequently, the SAIB DDS was stored at room temperature (RT) up to 14 days in a sealed blue cap bottle. For loading insulin into the SAIB DDS, insulin dry powder was added to the SAIB DDS under gentle stirring to a final concentration of 0.5% (w/w) after which the SAIB DDS was sonicated for 10 min to suspend the protein homogeneously.

Biosimilar mucus was prepared the day prior to experiment according to the method previously described by our group (Boegh et al., 2014, 2015). In brief, PAA (0.9% w/v) was dissolved in 10 mM HEPES buffer, and 1.3 mM calcium chloride and 1.0 mM magnesium sulfate added under magnetic stirring. Subsequently, mucin was added (5% w/v) and pH adjusted toward neutral pH using sodium hydroxide (NaOH). A mixture of polysorbate 80 (0.16% w/v), cholesterol (0.36% w/v), and PC (0.18% w/v) was added together with BSA (3.1% w/v) and pH adjusted to 7.4 with NaOH. Prior to use, the biosimilar mucus was stored overnight at 4 °C.

Labeling of insulin with ^123^I for SPECT/CT imaging was done by dissolving insulin (1 mg) in 800 µL 10 mM HCl. Then, 200 µL of 0.5 M monopotassium phosphate (KH_2_PO_4_) buffer (pH 5.0) was added to the solution. To 500 µL of the resulting solution, 300 µL of 0.2 M KH_2_PO_4_ buffer (pH 5.0) and 120 µL of Na-^123^I (109.2 MBq) were added. The reaction was started by addition of 10 µL of chloramine T solution (1 mg/mL). The reaction mixture was mixed well and left at RT. After 3 min, the reaction was stopped by addition of 10 µL of sodium thiosulfate (2.5 mg/mL) solution in ultrapure water. After 5 min, the reaction mixture was applied on an RP cartridge (Sep-Pac C_18_, Waters, Milford, MA), the cartridge was washed with 3 mL of deionized water to remove unreacted low molecular weight compounds and salt. The product was washed from the cartridge with 2 mL of 60% (v/v) acetonitrile containing 0.01% (v/v) HCl to give a mixture of insulin and radioactively labeled insulin (70 MBq). Then, solvents were removed in vacuum, and the residue was dissolved in 0.7 mL ethanol containing 0.1% (v/v) HCl, giving a radioactive yield of 64%. The radioactive purity was determined by HPLC (HP 1050, Agilent Technologies Inc., Palo Alto, CA), equipped with X bridge^TM^ C_18_ (5 µm, 4.6 × 150 mm, Waters, Milford, MA). The linear gradient for mobile phase B was set from 0 to 60% over 30 min with a flow rate of 1 mL/min. The mobile phase A consisted of 0.1% (v/v) TFA/99.1% (v/v) ultrapure water and B consisted of 0.1% TFA (v/v)/80% acetonitrile (v/v)/19.9% (v/v) ultrapure water.

Four buffers were prepared: 10 mM MES and SSIF, both pH 6.5. SSIF consisted of sodium taurocholate (3 mM), sodium phosphatidylcholine (SPC) (0.2 mM), maleic acid (19 mM), and sodium chloride (68 mM) in ultrapure water, as previously described (García-Díaz et al., 2015). The buffers were stirred overnight, and pH adjusted to 6.5. For *in vitro* experiments, a 10 mM HEPES HBSS buffer pH 7.4 was prepared by dissolving HEPES in HBSS (hHBSS) and a 10 mM MES HBSS buffer pH 6.5 (mHBSS). The buffers for *in vitro* experiments were further added 0.5% (w/v) BSA to offset nonspecific binding.

### Biophysical characterization of the SAIB DDS

#### Small deformation rheology

An ARES-G2 Rheometer (TA Instruments, New Castle, DE) equipped with a Peltier plate and truncated cone (1°, 20 mm from TA Instruments, New Castle, DE) was used for all rheological measurements. To prevent evaporation from the sample, a solvent trap cover was used. All tests were run within the linear viscoelastic region.

The effect of temperature on the elastic modulus (*G*′) of the SAIB DDS was evaluated using a temperature sweep from 5 to 50 °C (1 °C/min) using an angular frequency of 1 rad/s and an oscillatory stress of 1 Pa. To evaluate the effect of storage conditions, time and immersion in test media (10 mM MES and SSIF, pH 6.5) on the SAIB DDS, a frequency sweep was conducted with an increasing angular frequency from 0 to 10 rad/s and an oscillatory stress of 1 Pa. Stress sweep tests were measured with an increasing oscillatory stress from 0 to 500 Pa and an angular frequency set to 1 rad/s.

Mucoadhesion of the SAIB DDS to biosimilar mucus was evaluated using a slightly modified version of a previously described method (Hassan & Gallo, 1990; Ivarsson & Wahlgren, 2012). Due to limitations in sample amount, the cone-plate geometry was used. The samples were analyzed using continuous ramp flow with increasing shear rates from 0 to 25 s^–1^ sampling five points per decade. The instrument was set to automatically await temperature equilibrium at 37 °C before starting the measurement. The SAIB DDS and biosimilar mucus were prepared as described in the section preparation of test material and buffers. In addition, mixed samples in ratios of 2:1, 1:1, and 1:2 (v/v), biosimilar mucus:SAIB DDS were prepared on the day of analysis using magnetic stirring for 15 min.

#### Loss on drying

Loss on drying was determined according to monographs for loss on drying and sucrose, as described in the British Pharmacopoeia (British Pharmacopoeia, 2015). Briefly, the weight of the samples was measured before and after placing the samples in an oven at 105 °C for at least 24 h. Subsequently, the weight was measured again and the percentage loss of weight calculated.

#### Microstructure

The microstructure of the SAIB DDS was investigated both after 8 h of exposure to MES or SSIF buffer as well as before and after subjecting the SAIB DDS to the dynamic gastric model. The images were captured using a bright-field microscope using 100× magnification (Nikon Eclipse Ti-S, Tokyo, Japan), equipped with a Lumenera camera (Lumenera, Ottawa, Canada).

### Effect of gastric processing on integrity and insulin release

The dynamic gastric model experiments were performed according to previous studies (Vardakou et al., 2011; Wickham et al., 2012), using a constant volume of 50 mL, simulating fasted state *in vivo* conditions. The addition of simulated gastric juice was performed at a constant rate of 1 mL/min, and the dynamic gastric model was programed to eject 10 mL samples every 10 min. Fluid compositions were as follows: gastric acid solution: 58 mM NaCl, 30 mM KCl, 0.5 mM CaCl_2_, 0.86 mM NaH_2_PO_4_, and 20 mM HCl, pH 2. Since fasted state conditions including fasted state secretions of gastric juices were simulated, a flow rate of 1 mL of gastric acid solution was used. The experiments were performed by adding 2 g insulin-loaded SAIB DDS to the fundus/corpus compartment, followed by 40 min processing. Four samples were collected and analyzed for insulin content over this period, along with the 50 mL residual collected when terminating the experiment. Insulin release was quantified using RP-HPLC (Ultimate 3000, Dionex, Thermo Fisher Scientific, Waltham, MA) equipped with an Aeris Widepore XB-C18 column (100 × 2.1 mm, 3.6 µm, Phenomenex, Torrance, CA) and UV detection at 214 and 275 nm. The mobile phase A consisted of ultrapure water with 0.1% (v/v) trifluoroacetic acid (TFA), and the mobile phase B consisted of acetonitrile/0.1% (v/v) TFA. Insulin was eluted using a linear gradient of mobile phase B from 20 to 60% over 3.5 min at a flow rate of 0.8 mL/min and using a column temperature of 40 °C. The injection volume was 10 µL, and standard curves of insulin dissolved in the relevant medium were linear in the range of 0.5–500 µg/mL.

### Release behavior

The SAIB DDS was evaluated by adding 100 mg of the insulin-loaded SAIB DDS to 3 mL 37 °C MES or SSIF. The loaded SAIB DDS samples were maintained at 37 °C under constant linear shaking using a water bath (GLS400, Grant Instruments, Cambridge, UK). Sample volumes of 100 µL were collected after 0, 15, 30, 45, 60, 120, 180, 240, 300, 360, and 1440 min at 37 °C and replenishment with buffer was done. To prevent degradation of insulin after sampling, 5 µL 2% (v/v) formic acid was added to collected samples (Boegh et al., 2015). Finally, insulin release was quantified as described in the method for effect of gastric processing on integrity and insulin release.

### *In vitro* evaluation in the Caco-2 cell culture model

^3^H-mannitol was used as a paracellular epithelial permeability marker to assess the influence of SAIB DDS on the epithelial integrity using the human colon adenocarcinoma cell line; Caco-2 cells (American Type Culture Collection, Rockville, MD). The cells were cultured in DMEM medium supplemented with 10% (v/v) FBS, penicillin (100 U/mL), streptomycin (100 μg/mL), 1% (v/v) l-glutamine, and 1% (v/v) non-essential amino acids (DMEM+). Cells in T75 culturing flasks were kept at 37 °C in a 5% CO_2_ humidified atmosphere and passaged weekly. Passage numbers used were 25–31. The cells were seeded 20 days prior to the experiment by adding 0.5 mL cell suspension onto polycarbonate filter inserts (1.12 cm^2^ growth area, pore size of 0.4 μm) in a 12-well plate (Corning Costar, Tewksbury, MA) in a concentration of 8.9 × 10^–4^ cells/cm^2^. Culturing was done with 1 mL DMEM + in the well and 0.5 mL DMEM + in the insert and replacing this medium with fresh medium every other day.

To evaluate the effect of SAIB DDS on epithelial integrity and cell viability, the ^3^H-mannitol loaded SAIB DDS was applied to the cell monolayers in amounts of 50 µL or 150 µL at time 0, and supplemented with mHBSS buffer to a total volume of 400 µL. To prepare the loaded SAIB DDS, ^3^H-mannitol solution (1 μCi/mL in ethanol) was prepared and mixed with the SAIB DDS to allow for using a final concentration of 2.5 µCi/insert. The dosed volumes of the SAIB DDS were added to a scintillation vial in order to determine the exact ^3^H-mannitol donor starting concentration. Samples of 100 µL were withdrawn from the basolateral side of the monolayer every 15 min during the first hour and after that every 30 min for up to 4 h with replenishment of buffer after each sample withdrawal. Throughout the experiment, the cells were kept for 4 h at 37 °C, while placed on a benchtop orbital shaker (MaxQ2000, Thermo Fischer Scientific, Waltham, MA) set to 100 rpm. TEER was measured before and after the experiment after 15 min equilibration in hHBSS at RT, using a resistance chamber (Endohm-12, World Precision Instruments, Sarasota, FL) connected to a voltmeter (EVOM, World Precision Instruments, Sarasota, FL). As a control, 50 µL SAIB DDS was added to the cells and 370 µL mHBSS buffer containing ^3^H-mannitol was added. Samples of 100 µL were immediately collected from the apical side, and mixed by vortex with 2 mL scintillation fluid (Ultima Gold, PerkinElmer, Waltham, MA) in sealed vials prior to analysis using a Tri-Carb 2100 TR liquid scintillation analyzer (Canberra Packard, Dreieich, Germany).

Subsequent to the membrane integrity study, the effect on cell viability was evaluated. The cell monolayers were washed twice with hHBSS buffer pre-warmed to 37 °C and 320 µL MTS/PMS solution (0.24 mg/mL MTS, 4.8 μg/mL PMS in hHBSS) was added on the apical side of each monolayer. The cell monolayers were then kept for 1.5 h at 37 °C using the before mentioned benchtop orbital shaker set to 100 rpm. Subsequently, aliquots of 100 µL from each insert were transferred to a 96-well plate (Corning Costar, Tewksbury, MA) and the absorbance measured at 492 nm using a POLARStar plate reader (BMG LABTECH, Ortenberg, Germany).

### *In vivo* behavior visualized by SPECT/CT

Animal studies were approved by the Finnish National Animal Experiment Board (ESAVI/3631/04.10.03/2012) and performed in accordance with the Animal Welfare Act (247/1996), NIH Guide for Care and Use of Laboratory Animals and Good Laboratory Practices for Animal Research. Fischer albino male rats (F344/NCrHsd, Harlan, Netherlands, 236–252 g) were group-housed in a 12:12 h light/dark cycle. The rats had free access to rodent food and tap water.

The *in vivo* mucoadhesive potential of the SAIB DDS after oral administration was evaluated in rats using SPECT/CT imaging. Six rats were quarantined the minimum of 1 week under standard day/night lightening circles with free access to food and water at RT. The rats received either ^123^I-insulin in solution, ^123^I-insulin incorporated into SAIB DDS or ^123^I-insulin co-administered with the SAIB DDS using oral gavage. Depending on the yield of the labeling procedures, the rats received 12–41 MBq of activity in a total sample volume of 1 mL. 30–90 min prior to dosing the radiolabeled test systems; the rats received intra-gastric sodium iodine solution (250 µL, 10 mg/mL) to saturate the thyroid with iodine according standard to clinical practice (Notes for Guidance on the Clinical Administration, 2019). The rats were imaged with a four-headed small animal scanner (Mediso: Nucline^®^ NanoSPECT/CT, Bioscan, Washington, DC, manufactured and maintenance by Mediso, Budapest, Hungary) featuring 2.5 mm multi-pinhole rat apertures (Scivis, Göttingen, Germany). Imaging was performed under isoflurane anesthesia (2–3% v/v) in O_2_, and the body temperature was maintained warm using a heated animal bed (Equipement Vétérinaire Minerve, Esternay, France). Biodistribution of the radiolabelled test systems was followed for 22 h. After the last time point of imaging, the rats were sacrificed by cervical dislocation. Whole body SPECT images were collected in 16–20 projections using 20–30 s per projection resulting in a total acquisition time of 18 min. CT imaging was accomplished with 45 kVp tube voltage in 180 projections. For 3D co-registration and analysis, the SPECT images were reconstructed with HiSPECT NG software (Scivis, Göttingen, Germany) and fused with CT datasets by using the molecular imaging suite InVivoScope™ (Bioscan, Washington, DC).

### Statistical analysis

Statistical analysis was carried out in GraphPad Prism version 5.04 (GraphPad, La Jolla, CA) using one-way analysis of variance (ANOVA) with an alpha value of 0.05%, followed by Tukey’s posttest. Data are shown as mean ± standard deviation (S.D.). Unless otherwise stated, all tests were run in independent triplicates.

## Results and discussion

### Physiologically relevant temperature and immersion media enhance mucoadhesive behavior of SAIB

**Figure 1.**
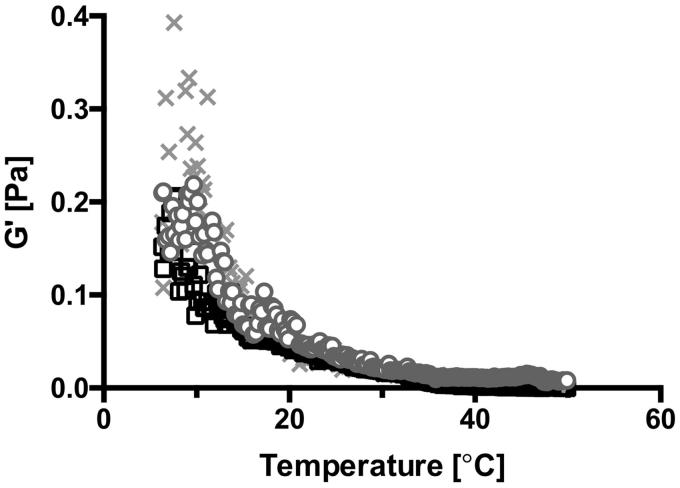
Temperature sweep. Data are plotted as triplicate samples of SAIB DDS (w/w) as a function of temperature.

When utilizing the SAIB DDS as a potential biomaterial, knowledge about its rheological properties is important, as they closely relates to drug release behavior (Szűts et al., 2008, 2010), spreading and adhesion on the mucosa, hence residence time *in vivo* (Carlfors et al., 1998; Edsman et al., 1998; Desai & Blanchard, 2000; Chang et al., 2002) and microstructural stability (Partal et al., 1997). At RT, the SAIB DDS is highly viscous, hence with a low spreadability. However, when increasing the temperature from 22 °C to 37 °C, temperature sweep profiles (Figure 1) demonstrated a fivefold decrease in the elastic modulus (*G*′), i.e. the hardness of SAIB DDS decreases from 0.04 Pa ± 0.01 to 0.008 Pa ± 0.004. This change in viscosity allows the SAIB DDS to spread on the surface of the GIT at body temperature, thereby facilitating a larger contact area between the SAIB DDS and the biobarrier.

**Figure 2.**
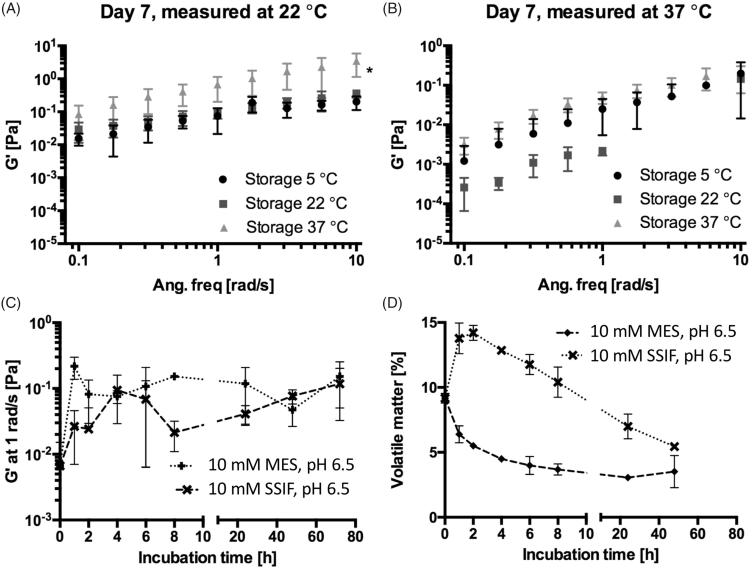
Effect of storage and incubation on the rheological profile of the SAIB DDS (A) and (B) frequency sweep of SAIB DDS stored at different temperatures and measured at either 22 °C (A) or 37 °C (B). (C) *G*′ after immersion of SAIB DDS in either 10 mM MES buffer or simulated small intestinal fluid (SSIF), both pH 6.5 and measured at 37 °C, and (D) loss on drying after exposure to the same buffer and temperature conditions as described for (C). Data are plotted as mean ± S.D.; *n* = 3.

A similar temperature dependency has been demonstrated for well-known bioadhesive biomaterials (Lin et al., 2012; Kong et al., 2015), and sucrose ester stabilized emulsions (Partal et al., 1997), explained by thermal agitation facilitating structural breakdown of the continuous phase, in this case being SAIB, hence decreasing the hardness of the material (Partal et al., 1997). To investigate the stability of SAIB DDS upon storage and at use, the rheological behavior was evaluated both at room and physiological temperature (22 and 37 °C) (Figure 2(A,B)) subsequent to storage at various temperatures (5, 22, and 37 °C) for up to 14 days (Figure 2(A,B) and supplementary). When running rheological analysis at 22 °C, SAIB DDS stored at 37 °C is characterized by a higher *G*′ compared to the SAIB DDS stored at lower temperatures (5 and 22 °C, Figure 2(A)) and considerably more stress was needed to cause a 10% decrease in *G*′ after storage at 37 °C, when comparing the samples tested (Supplementary data). This behavior is likely occurring due to irreversible coalescence or reversible deflocculation of the dispersed phase (alcohol), and eventually evaporation hereof into the continuous phase as a consequence of the increased storage temperature (Partal et al., 1997), confirming the data from Figure 1. Thus, when subjecting the destabilized system to a rapid temperature decrease from 37 to 22 °C, the dispersed phase is not reversed into its originally evenly distributed droplets. Instead, the continuous phase is forming a more rigid network resulting in an increased *G*′ (Partal et al., 1997). Accordingly, when increasing the measuring temperature from 22 to 37 °C, no difference was observed in the frequency (Figure 2(B)) as partial destabilization of the system has already occurred. Thus, SAIB DDS system was stored at 22 °C until further use.

As presence of biological fluids affects the rheological behavior of gel-based systems (Chang et al., 2002), the rheological properties of SAIB DDS were investigated during 72 h immersion in MES and SSIF at 37 °C (Figure 2(C)). Additionally, the amount of volatile matter (i.e. amount of substance evaporated) in SAIB DDS was determined after up to 48 h immersion in MES or SSIF buffer. From Figure 2(C), it is evident that immersion in MES or SSIF buffer (both pH 6.5) resulted in an increase of *G*′ as a function of time corresponding to a loss of volatile matter from the SAIB DDS already after 1 h immersion in buffer (Figure 2(D)); the same behavior as observed after 7 days storage at 37 °C (Figure 2(A)). This supports the previously stated hypothesis that irreversible coalescence of the ethanol phase is occurring. Interestingly, the increase in *G*′ at 1 rad/s and loss of volatile matter occurred in a delayed manner for the SAIB DDS immersed in the SSIF compared to MES buffer. It is previously reported that addition of lipophilic co-surfactants, such as the ones present in SSIF, can penetrate into the palisade layer of sucrose esters, hence induce swelling of the system (Rodriguez-Abreu et al., 2005). Such swelling allows for increased hydration of the SAIB DDS, thus increasing the percentage of volatile matter. This finding is highly interesting, as hydration of the delivery system is closely related to mucoadhesion, as well as to the diffusion of bioactive compounds through the intestinal mucosa (Peppas & Buri, 1985). SAIB DDS is thus believed to have highly interesting properties as DDS targeting the intestinal mucus due to high spreadability, swelling and consequently mucoadhesive behavior.

### SAIB displays considerable mucoadhesion to biosimilar mucus

One approach to increase the bioavailability of orally delivered biopharmaceuticals is to prolong the residence time, i.e. contact time of the drug with the mucosa at the relevant absorption site, which can be done by the development of a mucoadhesive delivery system (Ivarsson & Wahlgren, 2012). A commonly used method to assess the degree of mucoadhesion is to evaluate the potential changes in viscosity caused by the interaction between 1 and 3% (w/v) mucin dispersed in the biorelevant buffer and the delivery system of interest, using equation (1) (Hassan & Gallo, 1990; Oechsner & Keipert, 1999; Kesavan et al., 2010; Hägerström et al., 2012; Ivarsson & Wahlgren, 2012):

(1) $$\eta_{\text{adhesion}}=\eta_{\text{total}\;\text{system}}–\;\eta_{\text{mucin}\;\text{reference}}–\eta_{\text{delivery}\;\text{system}}$$

**Figure 3.**
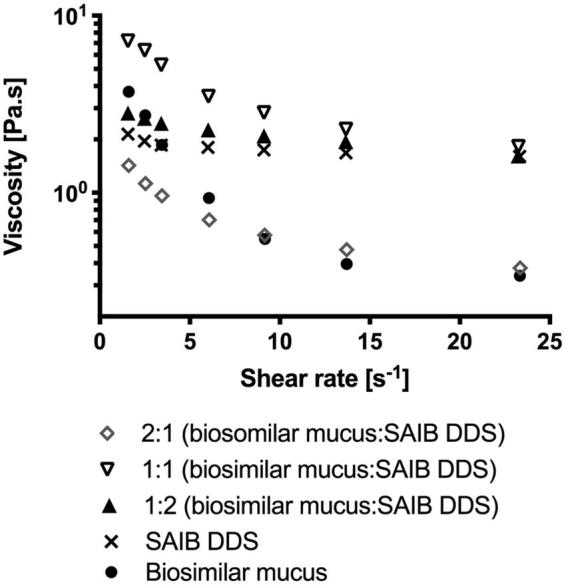
Continuous ramp flow with increasing shear rates from 0 to 25 s^–1^. Data are plotted as a representative sample chosen from triplicate measurements.

It has been shown, however, that a homogenized porcine mucus gel (2% w/w) serves as a better model compared to a mucin dispersion (Madsen et al., 1996a, 1996b). Yet, mucoadhesion cannot solely be ascribed to the formation of secondary bonds, such as the interaction between the hydrophobic backbone of the SAIB and the glycoprotein chains on the mucin. Mucus as a whole behaves like a steric and interactive barrier to diffusion of drugs (Madsen et al., 1996a, 1996b; Lai et al., 2009; Leileg & Ribbeck, 2011), for which reason we chose to implement a recently developed biosimilar mucus mixture containing 5% (w/v) mucin (Boegh et al., 2014, 2015) in the characterization of the mucoadhesion of the DDS (Figure 3). When comparing the biosimilar mucus with porcine intestinal mucus, a similar microstructure, as well as rheological properties, is observed (Boegh et al., 2014, 2015).

As mucoadhesion is affected by the SAIB concentration (Madsen et al., 1996a, 1996b), mixing ratios of 2:1, 1:1, and 1:2 (biosimilar mucus:SAIB, v/v) were used for the evaluation. When calculating *η*_adhesion_ at a shear rate of both 9 and 14 s^–1^ (both values well within the linear region of the viscosity profile) an increase in viscosity was observed for the ratio 1:1 (biosimilar mucus:SAIB, v/v) suggesting an interaction between the SAIB DDS and the biosimilar mucus. The interaction observed is likely primarily due to the formation of a high number of weak secondary bonds formed between the hydrophobic backbone of the SAIB and the mucin strands. Negative *η*_adhesion_ values were, however, obtained for the ratios 2:1 and 1:2. In those mixtures, the dominant phase was either SAIB or biosimilar mucus, and thus the network characterized being only the dominant phase, suggesting *η*_total system_ = *η*_delivery system_ for those mixtures, explaining the negative *η*_adhesion_.

For the mixing ratio of 2:1, biosimilar mucus being the dominant phase, the number of secondary bonds between biosimilar mucus and SAIB is lower when compared to the mixing ratio of 1:1, thus the mixtures resistance to deformation is not increased. This suggests that a local minimum concentration of the SAIB DDS is required to form significant interactions with the biosimilar mucus. At a ratio of 1:2, the network of the dominant phase (SAIB) is disrupted and smaller flow units are formed, thus decreasing the total viscosity of the mixture (Szűts et al., 2010). On the contrary, when equivalent volumes are present, no phase is dominating, and interactions are built, resulting in a positive *η*_adhesion_. Such eutectic behavior is known from, e.g. binary blends of fatty acids (Costa et al., 2009), where complete miscibility is only observed for equimolar ratios of fatty acids. What remains unanswered from this approach is, which ratio between biosimilar mucus and the delivery system best represents the actual *in vivo* situation. It is, however, shown that the interaction between SAIB DDS and the biosimilar mucus is possible given the optimal dosing volume.

### SAIB DDS remains stable and retains insulin within its matrix when subjected to gastric processing

**Figure 4.**
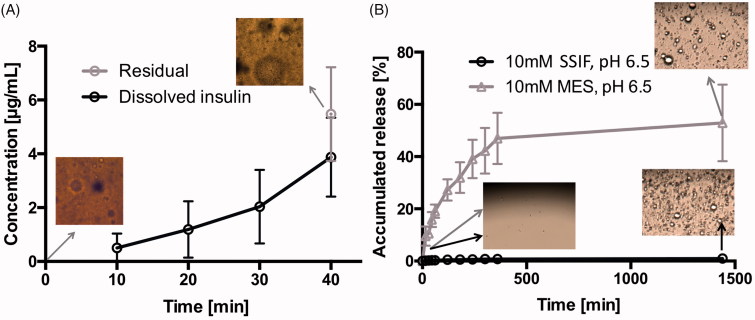
(A) Insulin release from SAIB DDS during the dynamic gastric model experiment simulating fasted state *in vivo* conditions. The image is obtained using light microscopy with a magnification of 20×. (B) Release of insulin at 37 °C from SAIB DDS after immersion in either 10 mM MES buffer or simulated small intestine fluid (SSIF), both pH 6.5. Data are plotted as mean ± S.D.; *n* = 3. The images are obtained using light microscopy with a magnification of 100×.

For an oral formulation, the stomach represents one of the first barriers the formulation must overcome for successful delivery. Therefore, the effects of physical shear stress in the antrum to the SAIB DDS was evaluated using the dynamic gastric model (Figure 4(A)). The dynamic gastric model is designed to simulate several unique and often disregarded features of the stomach, such as feedback regulated addition of acid and the physical shear forces of the antral compartment (Vardakou et al., 2011). Hence, the model is highly relevant for the evaluation of pharmaceutical oral dosage forms as it allows dosage form integrity, drug release and food effects to be studied *in vitro* (Wickham et al., 2012). Here, we examined insulin-loaded SAIB DDS (w/w) in the dynamic gastric model experiments simulating fasted state conditions. As shown in Figure 4(A), the observed concentration of insulin in solution gradually increased from 10 min to 40 min. After 40 min, the total insulin in solution corresponded to 20% of the theoretical content, e.g. 20% insulin released from the SAIB DDS in the stomach. Despite the release, the microstructure of the SAIB DDS remained intact (images, Figure 4(A)), suggesting that the SAIB DDS is capable of protecting insulin in the stomach and delivering the majority of insulin to the small intestine.

Furthermore, insulin release from SAIB DDS was evaluated in MES and the biologically relevant SSIF buffer, both pH 6.5 (Figure 4(B)). In MES buffer, an accumulated release of 52.9%±15 was observed, corresponding to 88.1 µg/mL ± 25. However, when submerging SAIB DDS into SSIF buffer, the accumulated release was only 1.5%±1, corresponding to 2.5 µg/mL ± 2. The significant difference in release when comparing the two buffers is caused by the observed swelling of the SAIB DDS matrix as a result of the lipophilic co-surfactants in SSIF buffer, as previously discussed. An increase in the hydrodynamic diameter of SAIB-based nanoparticles is previously shown to prevent diffusion through a poly-lactic acid membrane surrounding the SAIB based nanoparticles (Jølck et al., 2014). Thus, it can be speculated that the increased hydrodynamic diameter caused by swelling in the present study also explains the very limited insulin release observed in Figure 4(B). Moreover, the co-surfactants in the SSIF buffer might adhere to the surface of the SAIB DDS, thus preventing diffusion of insulin from the SAIB DDS matrix to the buffer.

### The SAIB gel system does not impair epithelial integrity and cell viability

**Figure 5.**
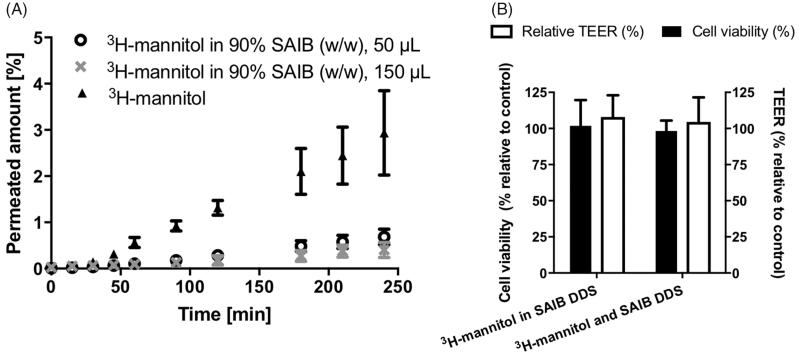
(A) Permeation of 3H-mannitol across a Caco-2 cell monolayer and permeation of 3H-mannitol when incorporated into SAIB DDS. (B) Cell viability (black bar) and epithelial integrity (open bar), assessed by transepithelial electrical resistance (TEER) of Caco-2 cell monolayers after application of SAIB DDS with either 3H-mannitol incorporated into SAIB DDS or co-administered with SAIB DDS for 4 h. Data are plotted as mean ± S.D.; *n* = 3.

Prior to conducting *in vivo* studies, the effect of SAIB DDS on the epithelial integrity and cell viability was evaluated using Caco-2 cell monolayers (Brøndsted et al., 1995) resembling intestinal epithelium. No effect was observed on the transepithelial electrical resistance (TEER) values across the monolayer exposed to the SAIB gel (108%±1 (S.D.) relative to control), proving that tightness hence integrity of the cell monolayer was intact. Furthermore, the results showed that addition of 50 µL SAIB DDS to the apical side of Caco-2 cell monolayers did not impair cell viability (104% ± 3 (S.D.) relative to control) (Figure 5(B)). Altogether, there was no indication that SAIB compromises the integrity of the epithelium. This finding is in agreement with reported long-term *in vivo* studies in rats, dogs, monkeys, and humans, showing no signs of toxic effect post oral administration of SAIB (Reynolds, 1998; Reynolds & Chappel, 1998; Food Additive Status List, 2002). In addition, it was revealed that the ethanol concentration used in the SAIB DDS did not have any effect on epithelial integrity and cell viability. Thus, it can be concluded that the tested SAIB DDS were compatible with the cell monolayers. The permeated amount of ^3^H-mannitol, included as a hydrophilic paracellular permeability marker, was not increased, but on the other hand considerably decreased when incorporated into the SAIB DDS (Figure 5(A)).

### SAIB DDS considerably increases intestinal residence time *in vivo*

The mucoadhesive behavior of orally administered SAIB DDS was evaluated in male F344/NCrHsd rats. Insulin, in the form of ^123^I-insulin was used as a model cargo for two purposes. First, the localization of the SAIB DDS encapsulating the molecule in the intestine, hence the mucoadhesive behavior could be assessed by SPECT/CT imaging. Second, the findings from the studies in the dynamic gastric model, suggesting that the SAIB DDS is capable of delivering the vast majority of the insulin to the small intestine target site could be verified.

**Figure 6.**
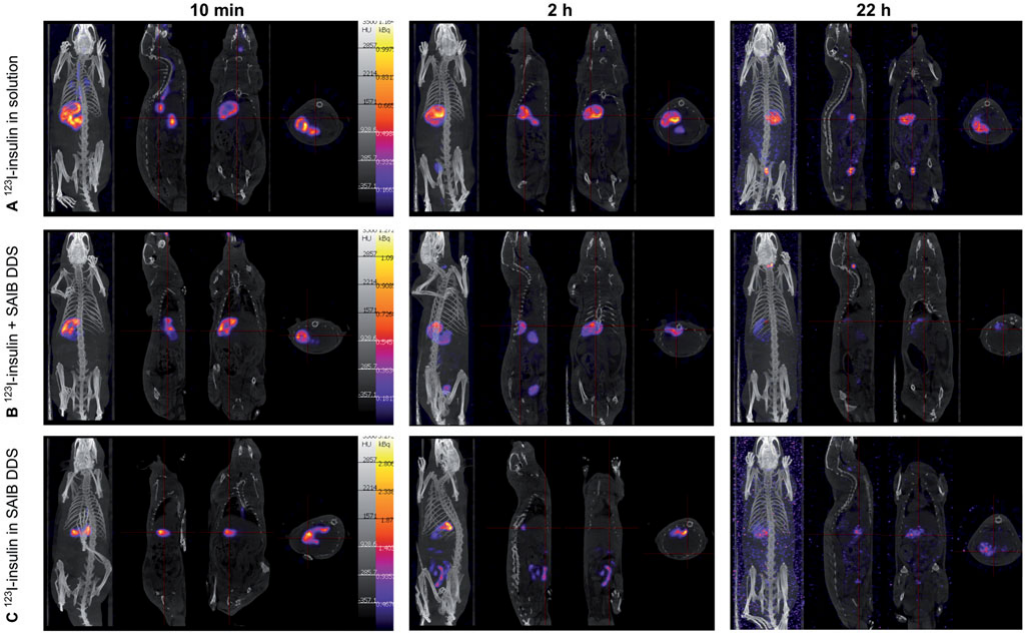
Representative SPECT/CT images showing the biodistribution of orally administered 123I-insulin 10 min, 2 h and 22 h after administration. The scale bar is the same for all images.

From Figure 6, it can be concluded, that the SAIB DDS clearly retained the radiolabel, likely still attached to the insulin, in the intestines for up to 22 h (Figure 6(C)). Contrary, orally administered ^123^I-insulin in ultrapure water alone did not show the presence of ^123^I-label in the intestines, whereas a ^123^I signal was primarily detected in the stomach. Two hours post administration, a fraction of the ^123^I-label was observed in the bladder, suggesting excretion of the ^123^I-label via the urine. Presence of ^123^I-activity in the animal cages after 22 h further supports this observation. It should of course be kept in mind that the radiolabel *might* be detached from the insulin during the experiment. Speaking against this is, though, that the distribution area of the radiolabel was much more confined for the ^123^I-insulin-loaded SAIB DDS as compared to the controls (^123^I-insulin alone and ^123^I-insulin co-administered with SAIB DDS), indicating that ^123^I-insulin remained encapsulated inside the SAIB DDS matrix. These findings are supported by the fact that only 1.5% insulin release was observed in SSIF and a similar tendency is expected *in vivo.* Moreover, a previous study showed the presence of SAIB in the intestine 24 h after oral administration (Phillips et al., 1976), supporting the conclusion that ^123^I is still entrapped in the SAIB DDS.

## Conclusions

In conclusion, we believe that SAIB is a very interesting biomaterial that can be applied to obtain mucoadhesion leading to increased intestinal retention *in vivo* as hypothesized. We base this conclusion on the combination of findings from thorough rheological assessment showing distinct mucoadhesion between the SAIB DDS and biosimilar mucus, and from *in vivo* studies revealing an increased intestinal residence time from 2 h to 22 h when comparing ^123^I-insulin in solution with ^123^I-insulin loaded in SAIB DDS. Moreover, both the dynamic gastric model and *in vivo* studies suggest, that SAIB DDS is capable of protecting its cargo against elimination after oral dosing in the stomach.

## Supplementary Material

Supplementary.docx
