# Supplementary material for: Exploring the mucoadhesive behavior of sucrose acetate isobutyrate: a novel excipient for oral delivery of biopharmaceuticals
Source: Drug Deliv. 2019 May 15;26(1):532–41. doi: 10.1080/10717544.2019.1606866 (PMC6534213; doi:10.1080/10717544.2019.1606866)
Supplement: Supplementary.docx [file IDRD_A_1606866_SM2301.docx]

**SUPPLEMENTARY**

**Effect of storage time on rheological behaviour**

*Figure S1. Frequency sweep of SAIB DDS stored at different temperatures and measured at either 22 °C (A) or 37°C (B). Data is plotted as mean ± s.d.; n=3.*

**Stress sweep for decrease in G’**

*Figure S2. Stress needed to cause a 10% decrease in G’. Data is derived from stress sweep test of SAIB DDS upon storage for 0, 7 and 14 d at 5, 22 and 37 °C. Closed bars correspond to measurements performed at 22 °C and open bars to measurements performed at 37 °C. Data is plotted as mean ± SD; N=1, n=3. An ARES-G2 Rheometer (TA Instruments, New Castle, DE, USA) equipped with a peltier plate and truncated cone (1 degree, 20 mm from TA Instruments, New Castle, DE, USA) was used. The rheometer was operated in stiff mode. Approximately 50 μL sample was placed in the center of the peltier plate and enclosed with a solvent trap cover. Stress sweep tests were measured with an increasing oscillatory stress from 0 to 500 Pa and an angular frequency set to 1 rad/s (N=1, n=3). Temperature was controlled at 22 and 37 °C, respectively.*
